# Research on an Equivalent Heat Source Model of the AC Arc in the Short Gap of a Copper-Core Cable and a Fire Risk Assessment Method

**DOI:** 10.3390/s24051443

**Published:** 2024-02-23

**Authors:** Yu Li, Rencheng Zhang, Kai Yang, Yufan Qi

**Affiliations:** Key Laboratory of Process Monitoring and System Optimization for Mechanical and Electrical Equipment, Huaqiao University, Xiamen 361021, China; liyu1995d@foxmail.com (Y.L.); yangkai1@hqu.edu.cn (K.Y.); qiyufan0130@163.com (Y.Q.)

**Keywords:** AC arc, MHD, EHS, fire risk, neural network, genetic algorithm

## Abstract

The magnetohydrodynamics (MHD) model of the alternating current (AC) arc is complex, so a simplified equivalent heat source (EHS) model can be used to replace the complex model in studying the AC arc’s thermal characteristics and cable fire risk. A 2D axisymmetric AC arc MHD simulation model in the short gap of a copper-core cable is established in this paper. The AC arc voltage and current obtained by the model are consistent with experiments. The AC arc’s heat source distribution obtained by the MHD model is fitted to obtain the heat source function *Q* of the AC arc. *Q* is divided into 16 independent segmented heat sources, and a correction matrix is constructed to optimize the segmented heat sources. A neural network and a genetic algorithm give the prediction model and the optimal correction matrix of the segmented heat source. The EHS model optimized by the optimal correction matrix can obtain a minimum temperature error of 5.8/4.4/4.2% with the MHD model in different AC arc peak currents 2/4/6 A. The probability of a cable fire is calculated by using AC arc’s optimized EHS model when different numbers of AC arcs are generated randomly in AC half-waves. The EHS model can replace the complex MHD model to study the thermal characteristics of AC arcs and quickly calculate the probability of a cable fire caused by random AC arcs.

## 1. Introduction

When a cable ages, grounding faults and operation damages occur in electrical equipment and lines, and the electrode gap breaks down and generates an arc due to the strong electric field. The generation of the arc will bring high temperatures to the local area, promoting the decomposition and combustion of the polyvinyl chloride (PVC) sheath in the cable, thus causing severe electrical fires [1,2,3]. The formation of an arc is coupled to multiple physical fields, including an electric field, temperature field, flow field, and a magnetic field, so the MHD model can be used to describe the arc [4]. However, the MHD model is complex and challenging to solve when studying the thermal characteristics of random AC arcs. Therefore, the arc EHS model can be used to replace the complex MHD model to study the thermal characteristics of AC arcs and the risk of a cable fire caused by random AC arcs.

The MHD model is mature and accurate in studying the magnetic field distribution, temperature distribution, and flow velocity of a conductive fluid. Zhang, Z.F. et al. [5] established a vacuum arc MHD model to research the change in arc appearance under an external axial magnetic field. Liu, X.J. et al. [6] used MHD to simulate the nitrogen-hydrogen mixture’s arc characteristics and the magnetic field’s arc root motion. Wang, D.Q. et al. [7] studied the effectiveness of self-induced ultrasonic control of the microstructure of 316 stainless steel based on MHD. Ji, Y. et al. [8] studied the rate of change in MHD angular rate sensors in a complex environment. Rehmet, C. et al. [9] established an MHD model for a three-phase AC arc and studied the influence of electrode size on the arc. Rau, S.H. et al. [10] established a 3D MHD model of the DC arc to study the influence of the arc on the photovoltaic system. In addition, the MHD model can also be used to study changes in the flow velocity, flow rate, voltage gradient, and temperature distribution of a conductive fluid [11,12,13,14,15,16,17].

The MHD model needs to solve many equations and consumes much time. Therefore, when studying the thermal characteristics of the arc, the EHS model can be used to replace the MHD model, which saves a lot of calculation time. Aissani, M. [18] and Azar, A.S. [19] proposed that direct current (DC) arc welding is equivalent to using a double elliptical heat source to describe the temperature field distribution of the welding arc. Ghosh, A. et al. [20,21] compared the welding arc to an ellipsoid, a double ellipsoid, and a conical heat source and considered that the ellipsoid heat source model was closest to the practical temperature distribution. García-García, V. et al. [22] proposed a moving ellipsoid heat source for the thermal simulation process of tungsten–inert gas-shielded arc welding. Mohanty, U.K. et al. [23] proposed an overlapping double ellipsoid heat source model to simulate the thermal behavior of the AC square wave arc welding process. Liu, K. et al. [24,25] equated the small-gap DC arc in the oil and gas pipeline to two heat sources: the arc ellipsoid and the Gaussian surface heat source. Li, Y. et al. [26] established a plasma EHS model based on the geometry of the molten pool to study the keyhole mode welding process.

In summary, the MHD model can be used to study the characteristics of a conductive fluid under different working conditions. In the study of the thermal characteristics of the arc, the heat generation of the arc can be equivalent to an EHS of ellipsoidal and conical shapes, which reduces the complexity of the MHD model [27,28]. However, most existing studies focus on constant heat sources such as DC arcs and welding arcs, while the heat generation of AC arcs changes all the time. There are few studies on the EHS of AC arc and the fire risk assessment method related to AC arc. Therefore, studying the EHS model of an AC arc is of great significance.

The main contributions of this paper are as follows:

(1) The heat source distribution characteristics of an AC arc between short electrode gaps.

An MHD model of the AC arc generated between the short gap of a copper-core cable in the air is established and verified by experiments. The AC arc MHD model obtains the heat source characteristics of the AC arc. In an AC half-wave of 0.01 s, the heat production rate of the AC arc increases before the peak time of 0.005 s and then decreases. The position near the tip of the electrode produces the most heat.

(2) The EHS model of AC arc between short electrode gaps.

According to the heat source distribution characteristics obtained by the MHD model, the EHS basic model of the AC arc is established. The basic heat source of the AC arc is divided into 16 independent heat sources, which are optimized by a correction matrix. A back propagation (BP) neural network trains a correction matrix to obtain a prediction model for the segmented heat source. Then, a genetic algorithm is used to find the optimal correction matrix, and the EHS model optimized by the optimal correction matrix can obtain the minimum temperature error of 5.8/4.4/4.2% with the MHD model in different AC arc peak currents of 2/4/6 A.

(3) Probability of a cable fire caused by random AC arcs.

Based on the EHS model of AC arc, the probability of a cable fire caused by randomly generated AC arc in different AC half-wave numbers is calculated. At the same AC half-wave number, the probability of fire increases with the increase in randomly generated AC arcs. In addition, the fire risk probability will also increase with the increase in the AC arc current.

## 2. MHD Models and Experiments

### 2.1. Geometric Model and Material Physical Parameters

**Figure 1:**
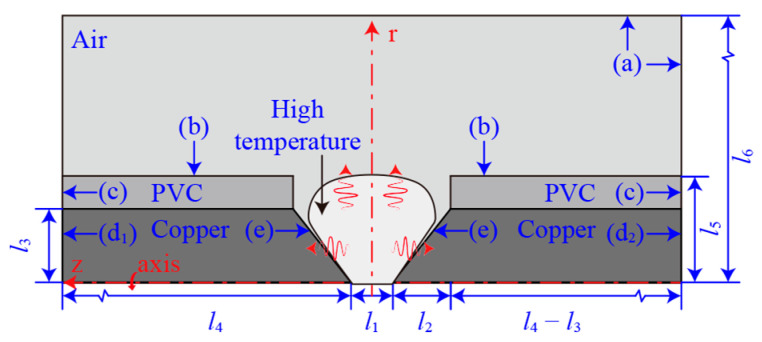
Cross section of AC arc geometric model of copper-core cable.

A 2D axisymmetric geometric model is established to study the AC arc generated by the short gap of copper-core cables, as shown in Figure 1. The geometric model includes air domain, copper wire, and PVC cable sheath. The end of the copper electrode is a conical tip, and the geometric model is connected to the 220 V/50 Hz AC source and a pure resistor in series between boundaries d_1_ and d_2_. The electrode gap *l*_1_ is 0.25 mm, the length of the electrode tip *l*_2_ is 1 mm, the radius of the copper electrode *l*_3_ is 1.38 mm, the length of single electrode *l*_4_ is 10 mm, the radius of the cable *l*_5_ is 2 mm, and the width of the whole geometric model *l*_6_ is 10 mm. An AC arc is generated between the electrode gaps, and the high temperature generated by the arc transfers heat to the surroundings.

The material parameters of copper and PVC required in the MHD model calculation are shown in Table 1. The density, specific heat capacity, dynamic viscosity, thermal conductivity, and radiation coefficient of air are functions related to temperature [17,24]. The current of the AC arc in the ‘zero current’ stage is 0, and it changes approximately sinusoidally during the arc burning stage. According to the characteristics of the AC arc ‘zero current’ stage in the subsequent AC arc experiments and reference [29,30,31], each ‘zero current’ time in the AC arc is set to be 0.0012 s in this paper, and the ‘zero current’ time is symmetrical at about the time when the AC voltage is 0. Therefore, in an AC half-wave, when the time *t* ≤ 0.0006 s or *t* ≥ 0.0094 s, the AC arc is in the ‘zero current’ stage, and the arc current *I_arc_* is 0. In addition to the rest of the time, the AC arc is in the burning stage, the *I_arc_* is expressed as *I_p_*·sin (100·π·*t*), and the *I_p_* is 2 A/4 A/6 A. The arc current is the input condition for solving the AC arc MHD model.

### 2.2. MHD Governing Equations and Boundary Conditions

An AC arc involves the coupling of multiple physical fields. In order to reduce the complexity of the model and speed up the calculation, the MHD model constructed in this paper introduces the following assumptions:

(1) The arc in the air is a plasma regarded as a continuous medium.
(2) The arc is an equilibrium plasma during equilibrium discharge, which is in a local thermodynamic equilibrium state.
(3) The arc plasma is a stable and incompressible fluid, and the flow of arc plasma is laminar.
(4) The arc burning time is very short, regardless of the reaction between the electrode and the arc’s erosion of the electrode contact.

According to the MHD theory, the mass of the AC arc plasma fluid will not change during the movement, so it satisfies the law of conservation of mass as shown in Equation (1). Because the arc plasma is electrically neutral, it is less affected by the electric field force. The generation time for arc plasma is short and less affected by gravity. Therefore, the momentum change rate of arc plasma is considered to be the sum of thermal pressure, viscous force, and Lorentz force. At the same time, the influence of the second viscosity is neglected. Equation (2) shows the law of conservation of momentum. The energy in the arc plasma is mainly composed of viscous dissipation, Joule heat, and radiation. Therefore, the entropy form of the energy conservation of the arc plasma satisfies Equation (3), where the viscous dissipation is Equation (4). The arc plasma is in equilibrium and satisfies the gas state equation shown in Equation (5) [16,25].

(1) $$\frac{d\rho }{dt}+\rho \nabla \cdot V=0$$

(2) $$\rho \frac{dV}{dt}=-\nabla p+\nabla \cdot \left(2\mu_{f}S\right)-\frac{2}{3}\mu_{f}\nabla \left(\mu_{f}\nabla \cdot V\right)+J\times B$$

(3) $$\rho T\frac{ds}{dt}=\Phi +\mu_{f}{\left(\nabla \cdot V\right)}^{2}+\lambda_{T}\nabla^{2}T+\frac{{\left(\nabla \times B\right)}^{2}}{\sigma \mu_{0}^{2}}-\varepsilon \sigma_{s}\left(T^{4}-T_{0}^{4}\right)$$

(4) $$\Phi =-\frac{2}{3}\mu_{f}{\left(\nabla \cdot V\right)}^{2}+2\mu_{f}S:S$$

(5) p=ρRT

where *ρ* is density, ***V*** is arc plasma flow velocity, *p* is pressure, *μ_f_* is viscosity coefficient, ***S*** is deformation tensor, ***J*** is current density, ***B*** is the magnetic induction, *T* is temperature, *s* is entropy, *Φ* is viscous dissipation term, *λ_T_* is thermal conductivity, *σ* is electrical conductivity, *μ*_0_ is magnetic permeability, *ɛ* is surface conductivity, *σ_s_* is the Steffen–Boltzmann constant, *T*_0_ is ambient temperature, *p*_0_ is one standard atmospheric pressure, and *R* is the gas constant.

The arc plasma is a conductor media, which satisfies Ohm’s law, Faraday’s law, and Ampere’s law, as shown in Equations (6)–(8), respectively. ***E*** is the electric field strength. Since the current density does not change with time, and magnetic induction intensity is a passive field, Equation (9) exists.

(6) $$J=\sigma \left(E+V\times B\right)$$

(7) $$\nabla \times E=-\frac{\partial B}{\partial t}$$

(8) $$\nabla \times B=\mu_{0}J$$

(9) $$\left\{\begin{array}{c} \nabla \cdot J=0\\ \nabla \cdot B=0 \end{array}\right.$$

The arc causes a large amount of heat to be created between the electrode gaps. Heat is transmitted through heat conduction to the surrounding air, the copper electrode, and the PVC skin, which can be expressed as Equation (10). During the arc’s ‘zero current’ stage and the AC half-wave without an arc, the heat generation *Q_arc_* is 0. There will also be convective and radiative heat transfer on the electrodes’ and PVCs’ surfaces. Surface heat dissipation *Q_sur_* is expressed as Equation (11).

(10) $$\frac{\partial (p_{m}c_{pm}T_{m})}{\partial t}-\nabla \cdot \left(k_{m}\nabla T_{m}\right)=Q_{arc}$$

(11) $$Q_{sur}=h\left(T_{sur}-T_{0}\right)+\varepsilon \sigma_{s}\left(T_{sur}^{4}-T_{0}^{4}\right)$$

where *ρ_m_* is density, *c_pm_* is heat capacity at a constant pressure, *k_m_* is thermal conductivity, *T_m_* is material temperature, *h* is the coefficient of convective heat transfer, *T_sur_* is surface temperature. The above physical parameters are related to the properties of the material itself.

This paper’s AC arc MHD model is calculated at one standard atmospheric pressure. The initial temperature is 293.15 K, the initial potential is 0 V, the initial magnetic vector potential is 0 Wb/m, and the initial velocity is 0 m/s. Table 2 shows the boundary conditions set in Figure 1. After that, the geometric model is meshed and Equations (1) through (11) are solved using COMSOL Multiphysics.

### 2.3. AC Arc Experimens

**Figure 2:**
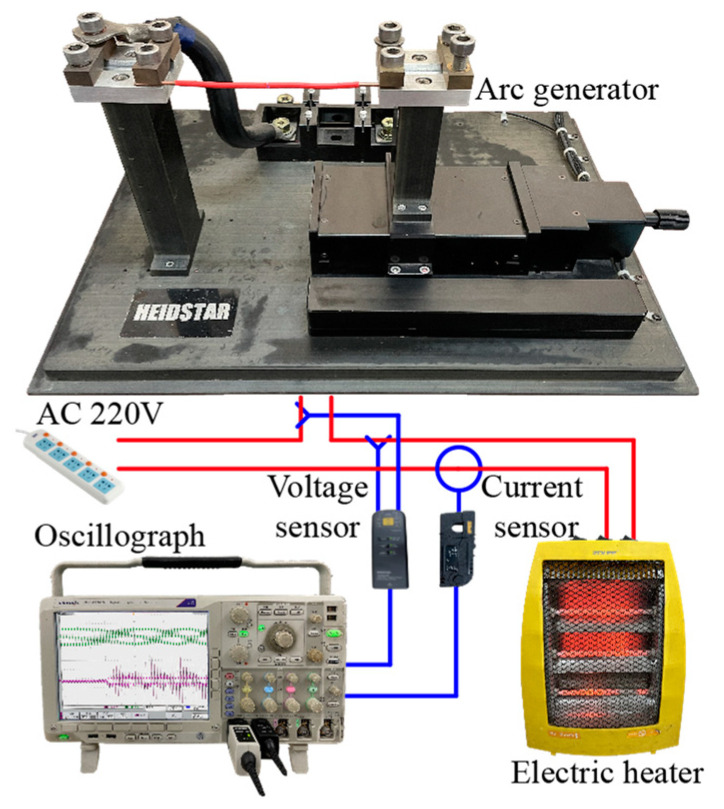
AC arc experimental platform.

An AC arc experimental platform was built for the studies presented in this paper, as shown in Figure 2. The controllable electrode gap in the arc generator is from Heidstar Technology (Xiamen, China) Co., LTD. The Keysight N2790A high-voltage differential probe and Keysight N2781B hall current sensor collect the AC arc voltage and current, respectively. The Tektronix DPO 4104B-L (Tektronix, Beaverton, OR, USA) digital phosphor oscilloscope stores and displays AC arc voltage and current. The heater is a pure resistive load, which can adjust the gear to produce an AC arc with a peak current of about 2 A/4 A/6 A.

**Figure 3:**
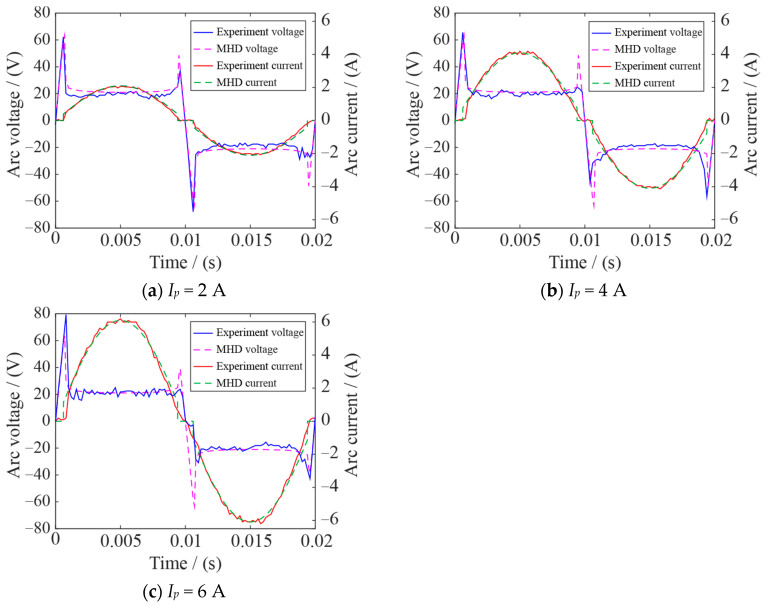
Comparison of AC arc voltage and current obtained by MHD model and experiments.

Figure 3 shows the arc voltage and current changes in the two adjacent AC half-waves obtained by the MHD model and the AC arc experiment. When entering an AC half-wave and at the end of an AC half-wave, the electrode gap is not broken down and is in the ‘zero current’ stage, the AC arc voltage is AC 220 V/50 Hz, and the AC arc current is 0. The arc voltage remains approximately unchanged after entering the stable burning stage of the AC arc. The arc voltage consists of anode voltage, arc column voltage, and cathode voltage. Because the AC arc studied in this paper belongs to a short arc, the arc column is short, so the arc column pressure drop is negligible. The anode voltage drop and cathode voltage drop are related to the electrode material and remain unchanged, so the AC arc voltage remains approximately unchanged during the burning stage [2,32,33]. In the AC arc burning stage, the arc current is approximately sinusoidal, and the amplitude is 2 A/4 A/6 A. Since the arc has a nonlinear pure resistance, the thermal power and electrical power of the arc are consistent [17,34]. Figure 3 shows that the changes in AC arc voltage and current obtained by the MHD model and AC arc experiment are relatively close, so the MHD model’s heat generation and temperature field distribution are considered to be accurate.

## 3. AC Arc EHS Model

### 3.1. Heat Source Distribution of AC Arc

**Figure 4:**
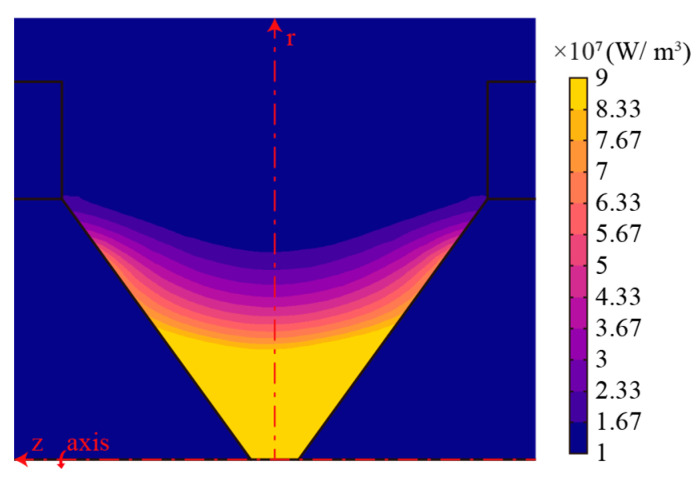
Heat source distribution between the electrode gaps when *I_p_* = 2 A, *t* = 0.001 s. (For easy display, the heat source range is selected from 1 × 10^7^ to 9 × 10^7^ W/m^3^, and the remaining range fluctuates wildly).

The MHD model of the AC arc can obtain the heat source distribution and temperature distribution within the AC arc. Figure 4 shows the heat source distribution case between the electrode gaps. The heat generation of the AC arc is mainly concentrated between the electrode gaps. The heat generation around the electrode gap and on the electrode is negligible. The heat generation near the electrode tip is much higher than that far away from the electrode tip.

**Figure 5:**
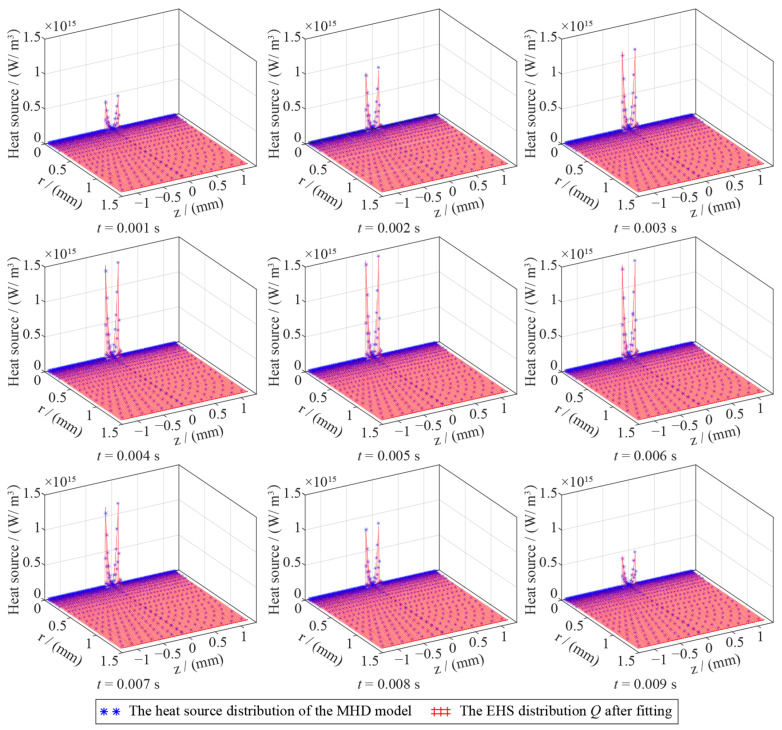
The MHD model obtained the AC arc heat source distribution and the AC arc heat source function obtained by fitting when *I_p_* = 2 A.

In an AC half-wave within an AC arc, heat generation changes with time. Figure 5 shows the heat source distribution case of the AC arc at different times obtained by the MHD model in an AC half-wave. The heat source of the AC arc is the highest at the electrode tip and decreases rapidly away from it, showing a ‘saddle’-shaped distribution. In addition, the maximum heat source at the tip of the electrode also changes with time, gradually increasing from 0.001 s to 0.005 s, reaching a peak at 0.005 s, and then decreasing, which is the same as the sine function of the AC half wave.

The distribution of the AC arc heat source obtained by the MHD model is fitted to *Q*, and it is considered that *Q* changes with three variables of *r*, *z*, and *t*. In Figure 4, with the increase in *r* on the electrode surface, the heat generation will decrease rapidly so that an exponential function can fit the heat source on the electrode surface. This paper considers that, when *r* is determined, the heat source is a parabolic function of *z* = 0 symmetrical distribution. Therefore, the heat source coordinates (*r*, *z*, *Q*) on the near electrode surface are fitted as (*r*, *l*_1_/2 + *l*_3_∙*r/l*_3_, *k*_1_∙e*^k^*^2^^∙*r*^), and the heat source at *z* = 0 is fitted as (*r*, 0, *k*_3_∙e*^k^*^4^^∙*r*^). Where *k*_1_, *k*_2_, *k*_3_, *k*_4_ are the coefficients needed for fitting and are related to time *t*. At different *I_p_* and *t*, the heat source distribution is fitted by Equation (12).

(12) $$Q\left(r,z,t\right)=\frac{k_{1}\cdot e^{k_{2}\cdot r}-k_{3}\cdot e^{k_{4}\cdot r}}{{\left(\frac{l_{1}}{2}+\frac{l_{2}\cdot r}{l_{3}}\right)}^{2}}\cdot z^{2}+k_{3}\cdot e^{k_{4}\cdot r}$$

**Figure 6:**
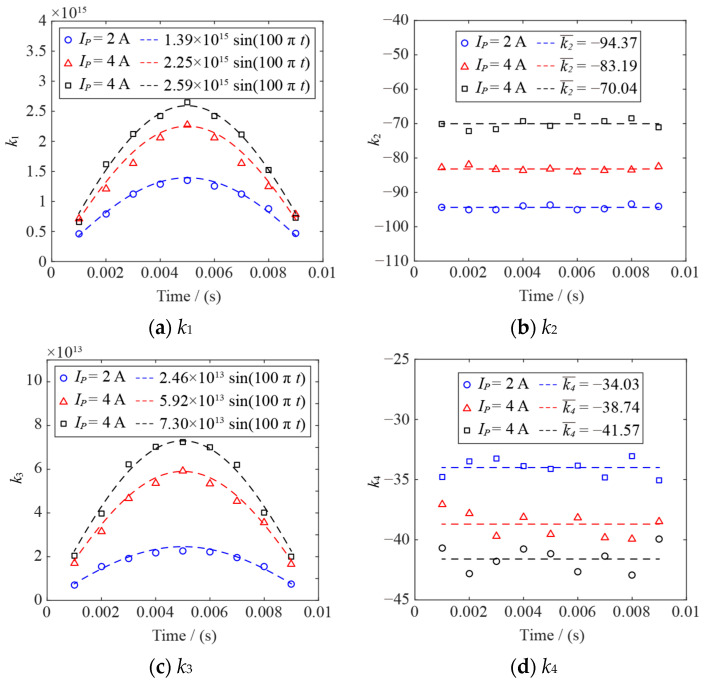
The variations in *k*_1_, *k*_2_, *k*_3_, and *k*_4_ in the EHS model *Q* of the AC arc are obtained by fitting the heat source distribution of the MHD model.

A case of comparing the AC arc EHS function after fitting Equation (12) with the heat source distribution obtained by the MHD model (*Ip* = 2 A) is shown in Figure 5. The difference between the fitted EHS function *Q* and the heat source obtained by the MHD model is slight. The coefficients *k*_1_, *k*_2_, *k*_3_, and *k*_4_ are obtained by Equation (12) in different AC arc peak currents and time, as shown in Figure 6. When the arc current is determined, *k*_1_ and *k*_3_ increase first and then decrease in an AC half-wave, consistent with the change in the sine function. Therefore, the time-dependent sine function can describe *k*_1_ and *k*_3_. When the AC arc peak current is determined, *k*_2_ and *k*_4_ change little with time. Therefore, the values of *k*_2_ and *k*_4_ are the mean values of *k*_2_ and *k*_4_. The relationships between *k*_1_, *k*_2_, *k*_3_, *k*_4_, and time are obtained, and the AC arc EHS model function *Q* is constructed.

### 3.2. Optimization of EHS Model

**Figure 7:**
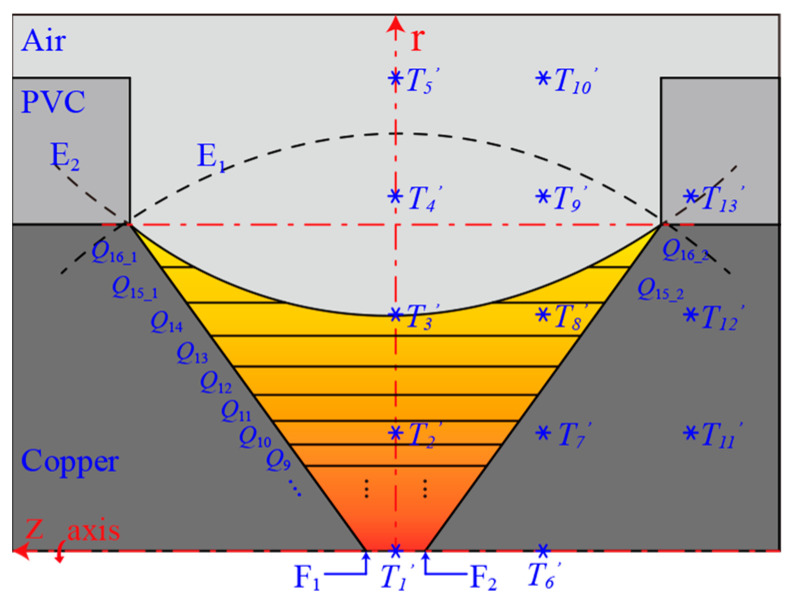
The boundary range of the EHS and the division into multiple independent EHSs.

According to the heat source distribution of the AC arc in Figure 4, the heat generation of the AC arc only occurs between the electrode gaps. In the study of the EHS model of the long arc, the heat source shape of the arc column region is considered to be a cylinder with a constant cross-sectional area. The AC arc studied in this paper belongs to the short arc category, so the arc column area is small, and the heat production area in the electrode gap is not a cylinder. This paper’s AC arc EHS model considers that the heat-producing area’s cross-sectional area gradually decreases from the electrode surface to the arc center. The heat generation region follows *Q*(*r*, *z*, *t*), and the range of the heat generation region is shown in Figure 7. In order to determine the heat generation boundary range of the EHS, an ellipse E_1_ (two electrode tips F_1_ and F_2_ as the focus.) is constructed. The ellipses E_2_ and E_1_ are symmetrical about *r* = *l*_3_, and E_2_ is used as the heat generation boundary of the EHS.

In order to further simplify the model and reduce the number of calculations, *Q*(*r*, *z*, *t*) is divided into 16 independent EHS in the *r* direction, and the *r* of each independent EHS is set to a fixed value. The dividing limits of the 16 independent EHSs are *r* = 0, 0.01, 0.03, 0.06, 0.1, 0.15, 0.21, 0.28, 0.36, 0.45, 0.55, 0.66, 0.78, 0.91, 1.05, 1.20, 1.38 mm, respectively. The intermediate value of the two adjacent partition boundaries is taken as the *r* of the independent heat source, so the values of *r*_1_, *r*_2_, …, *r*_16_ for the 16 EHSs are 0.005, 0.02, 0.045, 0.08, 0.125, 0.18, 0.245, 0.32, 0.405, 0.5, 0.605, 0.72, 0.845, 0.98, 1.125, 1.29 mm, respectively. Therefore, *Q*(*r*, *z*, *t*) is divided into *Q*_1_(*r*_1_, *z*, *t*), *Q*_2_(*r*_2_, *z*, *t*), …, *Q*_16_(*r*_16_, *z*, *t*), which are substituted as *Q_arc_* into Equation (10), and the temperature distribution can be obtained. The EHS *Q* of the AC arc is divided into multiple independent EHSs.

**Figure 8:**
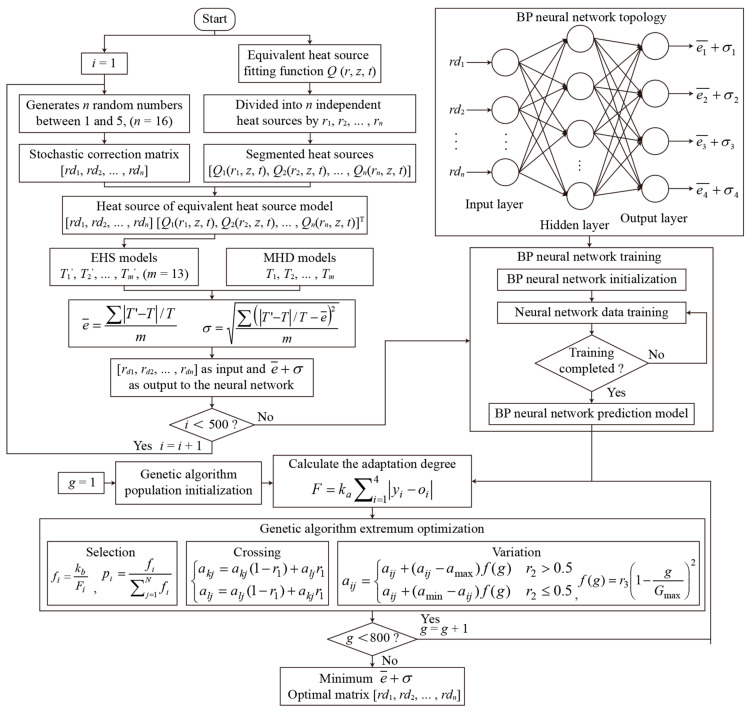
The correction matrix optimization process of AC arc-segmented EHS using neural network and genetic algorithm.

Since the EHS *Q*(*r*, *z*, *t*) is divided into independent segmented heat sources, the segmented EHS will lead to errors within the temperature calculation results of the AC arc MHD model. In order to reduce the temperature error, the segmented EHS needs to be optimized. Figure 8 shows the process of finding the optimal correction matrix using a neural network and genetic algorithm. Firstly, 16 random numbers *rd*_1_, *rd*_2_, …, *rd*_16_ between one and five are generated and used as random correction matrices. The segmented EHS optimized by the stochastic correction matrix is substituted into Equation (10) as *Q_arc_* to obtain the temperature distribution.

In Figure 7, 13 temperature measurement points *T*_1′_, *T*_2′_, …, *T*_13′_ are selected, and the same coordinate temperature measurement points *T*_1_, *T*_2_, …, *T*_13_ are selected in the AC arc MHD geometric model shown in Figure 1. The average relative error $\bar{e}$ between the temperature of the measurement points *T*_1′_ and *T*, and the overall standard deviation *σ* were calculated. $\bar{e_{1}}$ and *σ*_1_ are the average relative error and the overall standard deviation at 0.005 s (when one AC arc is generated). $\bar{e_{2}}$ and *σ*_2_ are the average relative error and the overall standard deviation at 0.015 s (when two AC arcs are generated continuously). $\bar{e_{3}}$ and *σ*_3_ are the average relative error and the overall standard deviation at 0.025 s (AC arcs are generated in the first and third AC half-waves, and no arc is generated in the second AC half-wave). $\bar{e_{4}}$ and *σ*_4_ are the average relative error and the overall standard deviation at 0.045 s (AC arcs are generated in the first, second, fourth, and fifth AC half-waves, and no arcs are generated in the second and third AC half-waves). Five hundred groups of random correction matrix are generated and used as the neural network’s input layer. The sum of the average relative error $\bar{e}$ and the overall standard deviation *σ* of the temperature measurement point is obtained by optimizing the segmented EHS with a correction matrix, which is used as the output layer of the neural network.

In this paper, a BP neural network is established to express the nonlinear relationship between the input layers and the output layers [35,36]. There are sixteen nodes in the input layer, eighteen nodes in the hidden layer, and four nodes in the output layer. The learning rate of the neural network is set to 0.1, and the training target is 0.00004. The neural network is trained to obtain the prediction model of the correction matrix.

A genetic algorithm optimizes the trained BP neural network. Compared with the traditional optimization algorithm, the genetic algorithm optimization has the advantages of group search, high search efficiency, operation speed block, and good adaptability. Figure 8 shows the process of the genetic algorithm, including population initialization, fitness function, selection operation, crossover operation, and mutation operation [37,38]. The first step is to initialize the population of the genetic algorithm. The individual coding method is real coding, which consists of four parts: ① the connection weight of the input layer and the hidden layer, ② the hidden layer threshold, ③ the connection weight of the hidden layer and the output layer, ④ the output layer threshold. The second step is to calculate the fitness. The absolute error value between the predicted output *y_i_* and the expected output *o_i_* is the individual fitness value *F*, where *k_a_* is the coefficient. The third step is the selection operation. This article uses the roulette method since the more negligible the fitness value *F_i_* of the individual *i*, the better. The fitness value is first inverted, then the probability *p_i_* of the individual *i* selected is calculated. Where *k_b_* is the coefficient, and *N* is the number of individuals in the population. The fourth step is cross-operation. Because the individual adopts real number coding, the cross-operation method adopts a real number crossover method. The *k*th chromosome *a_k_* and the *l* chromosome *a_l_* are crossed at the *j* position. Where *r*_1_ is a random number between 0 and 1. The fifth step is mutation operation. The *j*th gene *a_ij_* of the *i*th individual is selected for mutation. Where *a_max_* and *a_min_* are the upper and lower bounds of the gene *a_ij_*, respectively, *r*_2_ is a random number between 0 and 1, *g* is the current number of iterations, *G_max_* is the maximum number of iterations, and *r*_3_ is a random number. The sixth step is determining whether the maximum number of iterations is reached, and the optimal individual is output at the maximum number of iterations, creating an optimal AC arc-segmented heat source correction matrix [*rd*_1_, *rd*_2_, …, *rd*_16_], which can obtain the minimum temperature error with the MHD model.

**Figure 9:**
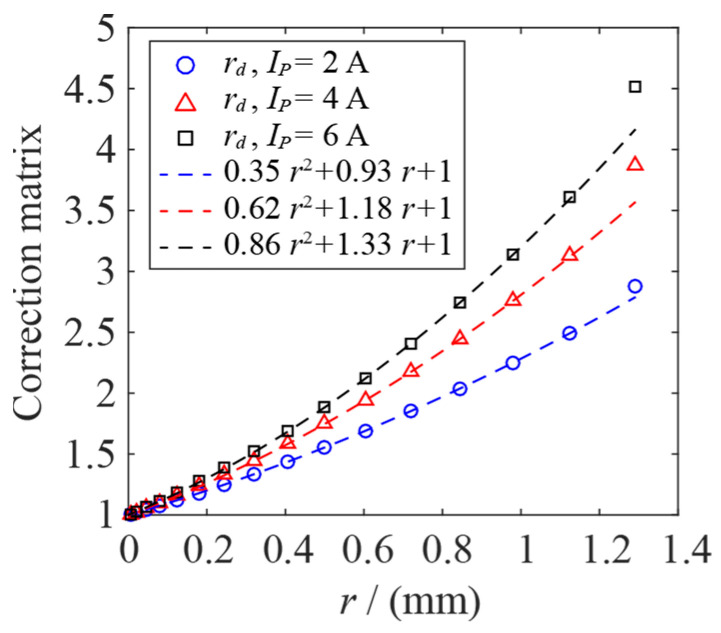
The optimal correction matrix of AC arc-segmented EHS.

Figure 9 shows the values of each element in the resulting optimal correction matrix and the fit with *r*. The optimized AC arc-segmented EHS calculates the temperature distribution and compares it with the MHD model. When *I_p_* is 2 A, 4 A, and 6 A, the average temperature error of the temperature measurement point is 5.8%, 4.4%, and 5.2%, respectively, and the overall standard deviation is 0.015, 0.011, and 0.013, respectively. Therefore, the EHS of the AC arc can be used to replace the MHD model in studying the thermal characteristics of the AC arc in the short gap of a copper-core cable.

**Figure 10:**
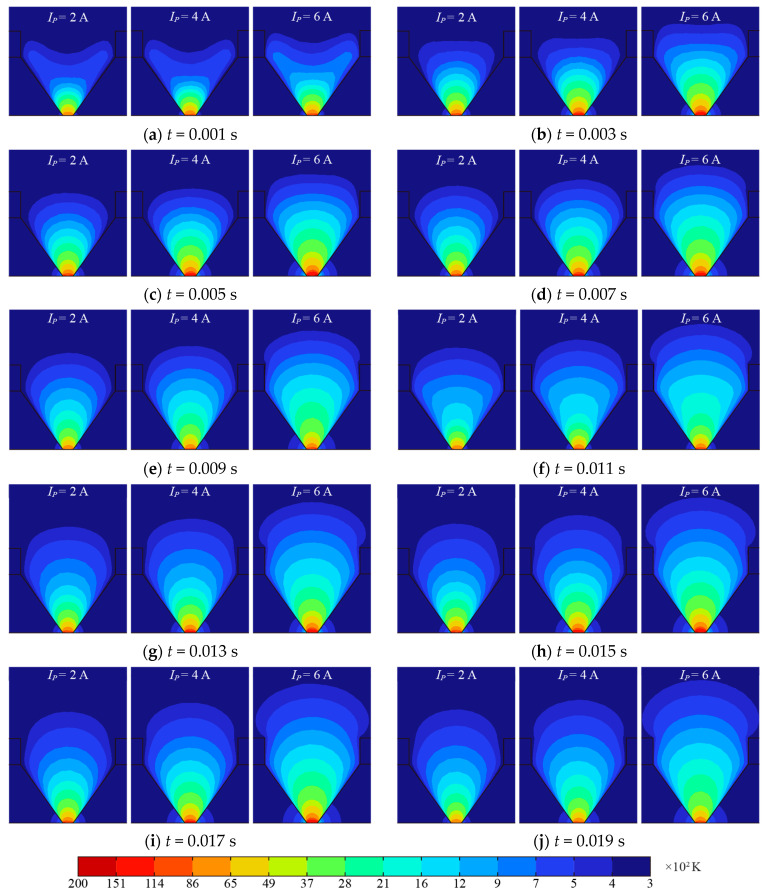
The temperature distribution of two consecutive AC arcs is obtained by using the EHS model.

Figure 10 shows the temperature distribution obtained using the segmented EHS of the AC arc when two AC arcs are generated continuously. In an AC half-wave, the high-temperature region exceeding 8000 K before the peak time of 0.005 s gradually increases and decreases after the peak time. However, during the heat generation process of the whole arc, the heat in the high-temperature region is gradually transferred to the surrounding area, and the temperature of the surrounding air and the electrode increases. After that, the second AC half-wave is generated, and the heat is accumulated at the residual temperature at the end of the first alternating current arc, prompting the temperature of the air and electrode to rise in a larger area. In addition, as the arc current increases, the electric power heat production increases, and the temperature will be higher in large arc currents.

## 4. Fire Risk Assessment for an AC Arc

AC arcs are randomly generated at different AC half-waves. The heat is accumulated in the AC half-wave with the arc, and the heat is dissipated in the AC half-wave without the arc. Therefore, the AC arc’s location difference will affect the heat accumulation. In addition, the AC arc’s current also affects heat generation. In the AC arc detection standard, the AC arc generated in a specific time is counted, and whether there is a fire risk is judged.

**Figure 11:**
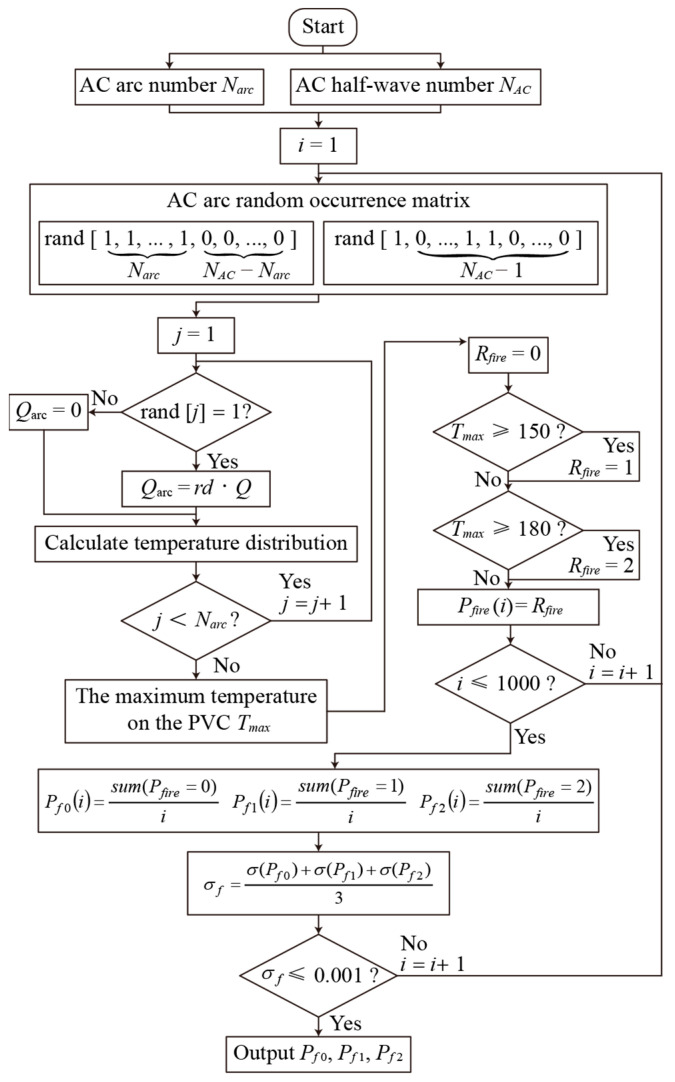
Process of calculating PVC fire risk level caused by random AC arc.

Figure 11 shows the fire risk assessment method and risk probability calculation process of the PVC cable fire caused by the AC arc. Based on the EHS model of an AC arc, this paper calculates the fire risk probability under different numbers of AC arc *N_arc_* generated at the random AC half-wave position when the AC half-wave number *N_AC_* is 10/20/30/40/50/60/70/80/90/100. The first half-wave of the AC half-wave number *N_AC_* produces an arc, and whether the subsequent *N_AC_*-1 half-wave produces AC arcs is random. The AC arc number *N_arc_* is less than or equal to the AC half-wave number *N_AC_*. In the AC half-wave of the AC arc, the *Q_arc_* is the EHS of the AC arc. On the contrary, the *Q_arc_* is 0. Then, the temperature distribution is calculated by Equation (10).

After that, the maximum temperature *T_max_* in the PVC calculation domain is obtained. If *T_max_* does not exceed 150 °C, it is considered that there is no fire risk, and the fire risk level *R_fire_* is recorded as 0. If the *T_max_* exceeds 150 °C, PVC will release HCL gas, which is considered to be a first-level fire risk, and the *R_fire_* is recorded as 1. If *T_max_* exceeds 180 °C, PVC begins to release smoke particles, considered a second-level fire risk, and *R_fire_* is recorded as 2.

**Figure 12:**
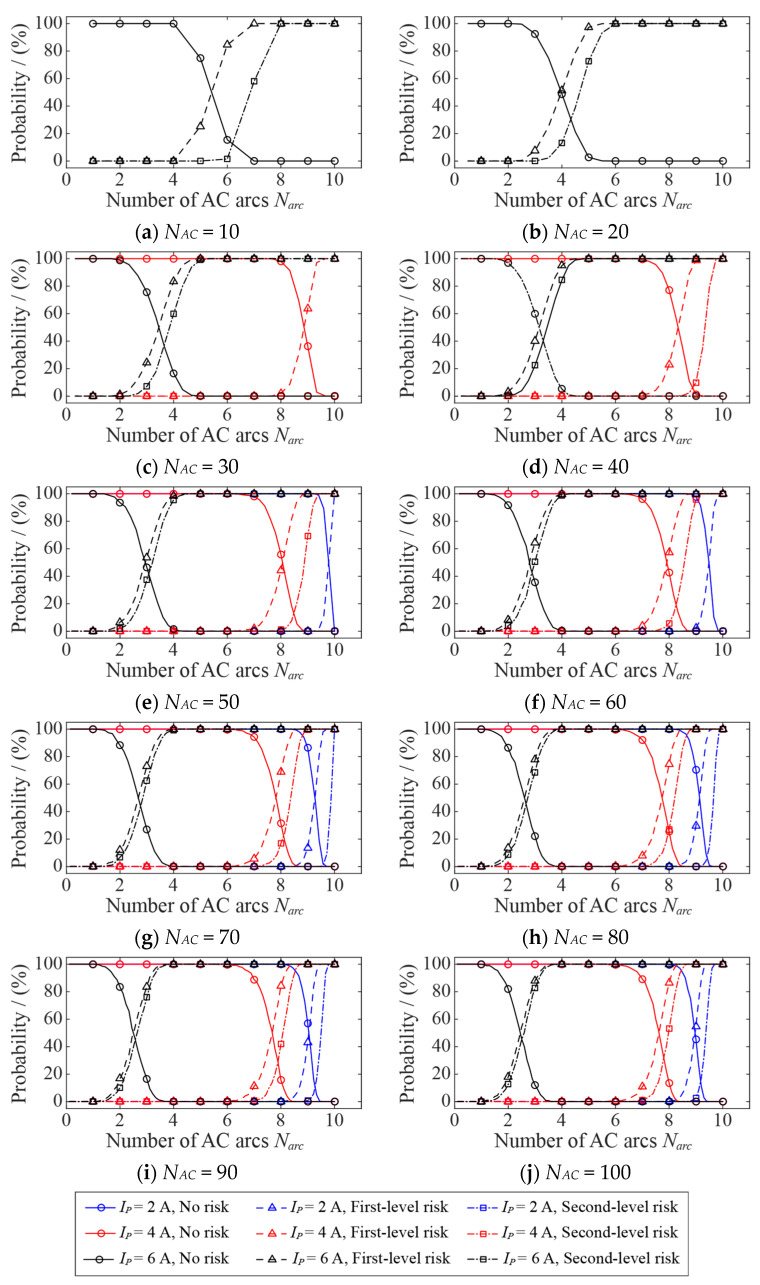
The PVC fire risk level probability of *N_arc_* AC arcs randomly generated in *N_AC_* AC half-waves. (When *I_P_* = 2 A, *N_AC_* = 10/20/30/40, and *I_P_* = 4 A, *N_AC_* = 10/20, there is no fire risk, which is not shown in the figure).

Because the generation of an AC arc is random and there are many groups in a random arrangement, it is necessary to calculate the fire risk level *R_fire_* of multiple groups of *N_AC_* and *N_arc_*. In the evaluation method set up in this paper, at least 1000 sets of data need to be calculated. The probability of *R_fire_* with different fire risk levels (*R_fire_* = 0/1/2) in all groups is calculated using the calculation process in Figure 11. *P_f_*_0_, *P_f_*_1_, and *P_f_*_2_ are the probability of no risk, first-level, and second-level fire risk, respectively. According to the theorem of large numbers, when there are enough groups to perform the calculation, the probability of each fire risk level tends to be fixed. Because PVC must produce HCL when it produces smoke, the probability of second-level fire risk is also included in first-level fire risk. Figure 12 shows the probability of no risk, first-level, and second-level fire risk when different AC arc numbers are randomly generated in different AC half-waves.

It can be seen from Figure 12 that, when the number of AC half-waves *N_AC_* is the same, the higher the number of randomly generated AC arcs *N_arc_*, the lower the probability of no fire risk, and the probability of first-level fire risk and second-level fire risk will gradually increase. At the same AC half-wave number and AC arc number, the probability of no fire risk decreases with the increase in arc current, and the probabilities of first-level fire risk and second-level fire risk increase with the increase in arc current. Different numbers of AC arcs are generated randomly in different AC half-waves, and the probability of fire risk is also different. A larger arc current and a more significant number of AC arcs will bring a higher fire risk. This study’s AC arc fire risk probability can provide a reference for AC arc detection standards and fire risk warnings.

## 5. Conclusions

This paper investigates the EHS model of AC arcs generated in the copper-core wire’s short gap and a fire risk level evaluation method. The research contents and results are as follows:

(1) An AC arc MHD model coupled with thermal, flow, electric, and magnetic fields is constructed. The AC arc voltage and current obtained by the AC arc experiment are compared with the AC arc MHD model to prove its correctness. In an AC half-wave, the heat production in the electrode gap increases first and then decreases. The heat production near the electrode tip is much higher than that around it.
(2) The heat source distribution obtained by the AC arc MHD model is used to obtain the EHS *Q* of the AC arc through fitting. The EHS *Q* is divided into 16 AC arc-segmented heat sources, and a correction matrix is constructed to optimize the AC arc EHS model. A BP neural network and a genetic algorithm obtain the optimal correction matrix of the AC arc’s segmented EHS model. The optimized EHS model of the AC arc is used to calculate the thermal characteristics of the AC arc, which can obtain the temperature errors of 5.8/4.4/4.2% when the MHD model in AC arc peak currents are 2/4/6 A. The calculation time of the MHD model can be significantly reduced by using the EHS model of AC arc with the double tips’ short gap proposed in this paper.
(3) The EHS model of an AC arc is used to calculate the probability of PVC fire risk (no fire risk, first-level fire risk, second-level fire risk) caused by a random number of AC arcs generated in different AC half-wave numbers. It is worth noting that there is no fire risk when the number of AC arcs is small. With the same AC half-wave numbers, the probability of no fire risk decreases with the increase in the number of AC arcs and the arc current, and the probability of first-level fire risk and second-level fire risk increases with the increase in the number of AC arcs and the arc current. The fire risk probability provided in this paper can be used to judge the fire hazard caused by the AC arcs to the cable.
(4) This study provides a research method for the AC arc EHS model and a method of AC arc fire risk assessment. The EHS model replaces the MHD model to calculate the arc’s temperature distribution between the cable’s short gaps quickly and accurately. The cable fire risk probability of random AC arcs can provide a reference for formulating AC arc detection standards and preventing electric fires.

## Figures and Tables

**Table 1:** The material parameters of copper and PVC.

Parameters	Value
Density of PVC/(kg/m^3^)	5.0 × 10^2^
Heat capacity at constant pressure of PVC/[J/(kg·K)]	1.0 × 10^3^
Thermal conductivity of PVC/[W/(m·K)]	0.16
Surface emissivity of PVC	0.95
Conductivity of PVC/(S/m)	0
Density of copper/(kg/m^3^)	8.94 × 10^3^
Heat capacity at constant pressure of copper/[J/(kg·K)]	3.85 × 10^2^
Thermal conductivity of copper/[W/(m·K)]	4.0 × 10^2^
Surface emissivity of copper	0.5
Conductivity of copper/(S/m)	5.998 × 10^7^

**Table 2:** Boundary conditions settings.

Boundary	Temperature *T/*(K)	Pressure *P/*(atm)	Flow Velocity *v/*(m/s)	Magnetic Vector Potential *A/*(Wb/m)
a	$\frac{\partial T}{\partial n}=0$	1	$\frac{\partial v}{\partial n}=0$	0
b, e	Equation (11)	$\frac{\partial p}{\partial n}=0$	0	$\frac{\partial A}{\partial n}=0$
c, d_1_, d_2_	*T* = *T*_0_	$\frac{\partial p}{\partial n}=0$	0	$\frac{\partial A}{\partial n}=0$

## Data Availability

The data that support the findings of this study are available from the corresponding author upon reasonable request. The data are not publicly available due to the nature of this research.
